# Pain Relieving and Neuroprotective Effects of Non-opioid Compound, DDD-028, in the Rat Model of Paclitaxel-Induced Neuropathy

**DOI:** 10.1007/s13311-021-01069-8

**Published:** 2021-07-26

**Authors:** Laura Micheli, Raghavan Rajagopalan, Elena Lucarini, Alessandra Toti, Carmen Parisio, Donatello Carrino, Alessandra Pacini, Carla Ghelardini, Parthasarathi Rajagopalan, Lorenzo Di Cesare Mannelli

**Affiliations:** 1 Department of Neuroscience, NEUROFARBA-Pharmacology and Toxicology Section, University of Florence, Psychology, Drug Research and Child HealthViale Pieraccini 6, 50139 Florence, Italy; 2 Daya Drug Discoveries, Inc, St. Louis, MO 63121 USA; 3 Department of Experimental and Clinical Medicine, Anatomy and Histology Section, University of Florence, Largo Brambilla 3, 50134 Florence, Italy

**Keywords:** DDD-028, Glial cell, Neuroprotection, Nicotinic receptor, Oxidative stress, Paclitaxel

## Abstract

Chemotherapy-induced neuropathy (CIN) is a major dose-limiting side effect of anticancer therapy that can compel therapy discontinuation. Inadequate analgesic efficacy of current pharmacological approaches requires the identification of innovative therapeutics and, hence, the purpose of this study is to conduct a preclinical evaluation of the efficacy of DDD-028, a versatile pentacyclic pyridoindole derivative, against paclitaxel-induced neuropathic pain. In two separate experiments, DDD-028 was administered per os acutely (1–25 mg kg^−1^) or repeatedly (10 mg kg^−1^) in paclitaxel-treated rats. The response to mechanical noxious stimulus (paw pressure) as well as to non-noxious mechanical (von Frey) and thermal (cold plate) stimuli was investigated. Acute administration of DDD-028 induced a dose-dependent anti-neuropathic pain effect in all tests performed. Further, repeated daily treatment for 18 consecutive days (starting the first day of paclitaxel administration) significantly reduced the development of pain over time without the development of tolerance to the anti-hyperalgesic effect. Ex vivo analysis showed that DDD-028 was able to reduce oxidative damage of dorsal root ganglia as evidenced by the increase in the level of carbonylated proteins and the decrease in catalase activity. In the lumbar spinal cord, periaqueductal gray matter, thalamus, and somatosensory cortex 1, DDD-28 significantly prevented the activation of microglia and astrocytes. The pharmacodynamic study revealed that the pain-relieving effects of DDD-028 were fully blocked by both the non-selective nicotinic receptor (nAChR) antagonist mecamylamine and by the selective α7 nAChR antagonist methyllycaconitine. In conclusion, DDD-028 was active in reducing paclitaxel-induced neuropathic pain after single or repeated administrations without tolerance development and displaying a double symptomatic and neuroprotective profile. DDD-028 could represent a valuable candidate for the treatment of CIN.

**Supplementary Information:**

The online version contains supplementary material available at 10.1007/s13311-021-01069-8.

## Introduction

The success of cancer treatments is frequently limited by neuropathies which represent a major health concern [1]. Neuropathy resulting from chemotherapy can be disabling, causing a significant functional loss and decreasing the quality of life. Moreover, neuropathy is the predominant reason for dose modification and discontinuation of treatment, and may thereby affect overall survival [1]. Paclitaxel is an antineoplastic agent originally derived from the bark of the western yew tree, *Taxus brevifolia*, and one of the most effective and widely used drug in several solid tumors, including neck, lung, breast, head, and ovarian cancers and AIDS-related Kaposi’s sarcoma [2–8]. Unfortunately, paclitaxel induces sensory peripheral neuropathy characterized by burning pain symptoms, allodynia, hyperalgesia, tingling, and numbness. Neuropathy is positively correlated with increasing number of paclitaxel doses per cycle, total cumulative dose, and duration of infusion and can persist for months or years following the cessation of treatments [2]. Grade 3 or 4 sensory neuropathy occurs in 20–35% of patients receiving 250 mg/m^2^ paclitaxel every 3 weeks [3]. Several attempts have been made to treat or prevent CIN with various neuroprotective drugs, but the results are contradictory and most of them are either ineffective or caused adverse effects such as nausea, reflex dysfunctions, treatment-emergent nervousness, insomnia, tremor, anorexia, or stomach burning [4–9]. Commonly used anti-neuropathic treatments, such gabapentin, lamotrigine, or pyridoxine plus pyridostigmine, have not shown efficacy in random clinical trials [4]. Amitriptyline or oxycodone only diminishes the pain symptom associated with taxane-induced neuropathy. Therefore, it is crucial to identify novel, safe therapeutic strategies that may efficiently prevent or suppress both the painful condition and damage to the nervous system.

**Fig. 1:**
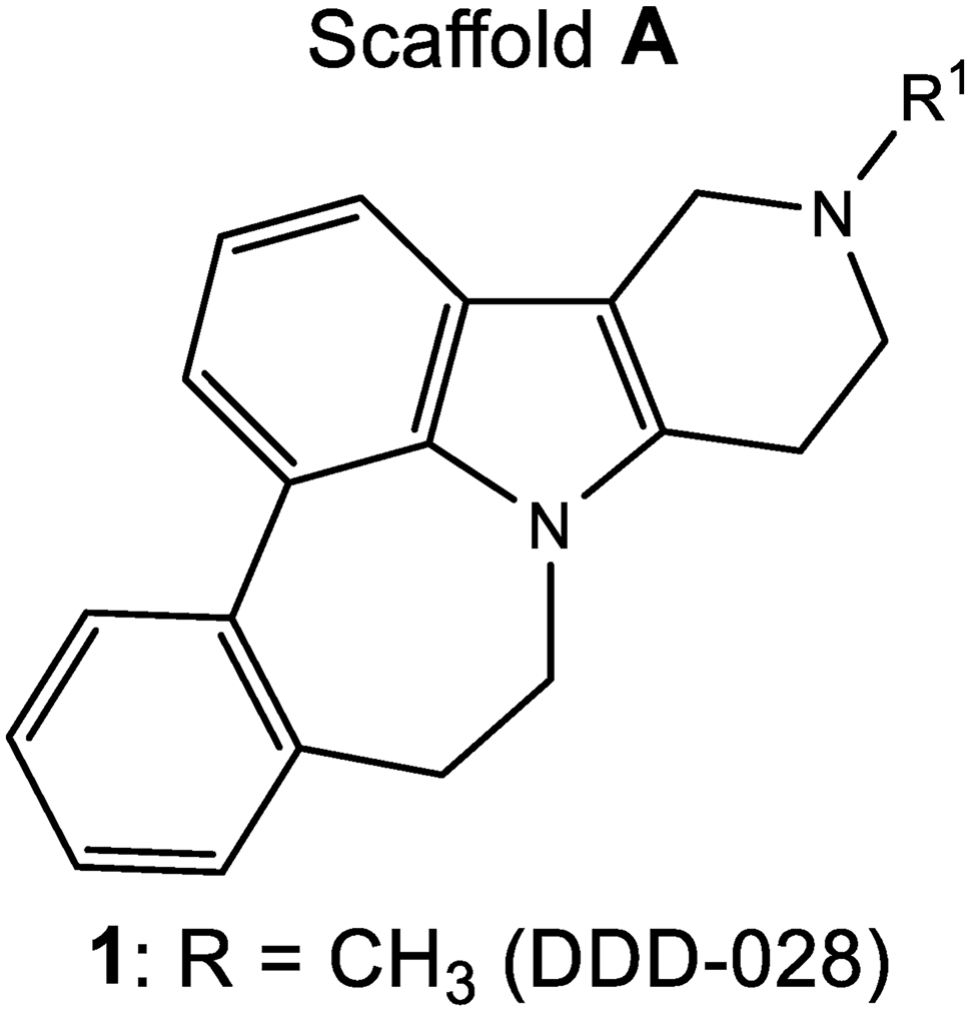
Structure of DDD-028

Accordingly, our extensive work on pentacyclic pyridoindole heterocycles such as scaffold A (Fig. 1) [5–8] resulted in the identification of a potent, non-opioid analgesic, DDD-028, for the potential treatment of CIN. The chemical properties of DDD-028 are summarized in Table 1 [9–11]. The purpose of the study was to evaluate the effect of acute and sub-chronic administrations of DDD-028 on paclitaxel-induced neuropathic pain in rats. The relief of pain hypersensitivity as well as the protective effects on the peripheral and central nervous system along with the pharmacodynamic profile will be presented herein.

**Table 1:** Chemical and preliminary safety properties of DDD-028

Parameter	Value
Molar mass	288.16 g/mol
clogP [11]	3.27
H-Bond donors	1
H-Bond Acceptors	0
Estimated pK_a_ [9]	8.1
Polar surface area [10]	6.8 Å^2^
CNS MPO score [10]	4.5
hERG binding	3.4 nM
Mini-Ames	Negative
C_max_	1.9 ng/mL
T_max_	2.0 h
Half-life	3.5 h

## Materials and Methods

### Animals

For all the experiments described below, male Sprague–Dawley rats (Envigo, Varese, Italy) weighing approximately 200–250 g at the beginning of the experimental procedure were used. Animals were housed in CeSAL (Centro Stabulazione Animali da Laboratorio, University of Florence) and used at least 1 week later after their arrival. Four rats were housed per cage (size 26 × 41 cm^2^), kept at 23 ± 1 °C with a 12-h light/dark cycle, light at 7 a.m, and were fed with standard laboratory diet and tap water ad libitum. All animal studies were carried out according to the Directive 2010/63/EU of the European parliament and of the European Union council (22 September 2010) on the protection of animals used for scientific purposes. The ethical policy of the University of Florence complies with the Guide for the Care and Use of Laboratory Animals of the US National Institutes of Health (NIH Publication No. 85–23, revised 1996; University of Florence assurance number: A5278-01). Formal approval to conduct the experiments described was obtained from the Italian Ministry of Health (No. 498/2017-PR) and from the Animal Subjects Review Board of the University of Florence. Experiments involving animals have been reported according to ARRIVE guidelines [12]. All efforts were made to minimize animal suffering and to reduce the number of animals used.

### Paclitaxel Rat Model of Neuropathy

Paclitaxel was dissolved in a mixture of 10% saline solution and Chremophor EL, a derivative of castor oil and ethylene oxide that is clinically used as paclitaxel vehicle. Rats were injected intraperitoneally (i.p.) with paclitaxel (2.0 mg kg^−1^) on four alternate days (days 1, 3, 5, and 8) [13, 14]. Control animals received an equivalent volume of the vehicle.

### DDD-028 Administration and Study of the Pharmacodynamic Mechanisms

To evaluate its symptomatic efficacy, DDD-028 [5–8] was suspended in 1% carboxymethylcellulose (CMC) and acutely administered per os with the dose ranging from 1 to 25 mg kg^−1^ on day 10, when paclitaxel neuropathy was well established. Thereafter, to demonstrate a protective effect, repeated per os administrations of DDD-028 10 mg kg^−1^ were carried out daily from the beginning of the paclitaxel administration (day 1) to the end of the experiment (day 18). Control animals were treated with vehicles.

To determine possible pharmacodynamic mechanisms, the following compounds were administered along with DDD-028: pan nicotinic receptor antagonist mecamylamine (MECA; 2 mg kg^−1^, i.p.) [15, 16], selective α7 nAchR antagonist methyllycaconitine (MLA; 6 mg kg^−1^, i.p.)[17], Kv7 channel blocker XE991 (1 mg kg^−1^, i.p.) [18, 19], and σ_1-_σ_2_ agonist (method described below). For MECA, 2 protocols were adopted: in the first, a single dose of MECA was administered 15 min before DDD-028 injection, an in the second, two doses of MECA were given, 15 min before DDD-028 and 45 min after DDD-028 treatment. MLA and XE991 were administered once, 15 min before DDD-028 treatment. Control animals were treated with vehicles. To assess the effect of σ_1_/σ_2_ receptors on the analgesic activity of DDD-028, the σ modulation of movements and posture were used. It is known that σ receptors are concentrated in brain structures that control movement, such as the red nucleus and substantia nigra. The unilateral microinjection of σ receptor agonists, such as 1,3-di-(2-tolyl)guanidine (DTG), ( +)-N-allylnormetazocyne [( +)-SKF-10,047], and ( +)-3-(3-hydroxyphenyl)-N-(1-propyl)piperidine [( +)-3-PPP], into the red nucleus induces neck dystonia in rats. The antidystonic effect observed in rats was shown to be related to σ receptor antagonism [20]. The neck dystonia was quantified by measuring the torsion of the neck. Briefly, the torticollis was quantified by measuring the torsional deviation of the head from the horizontal plane, using the eyes of the animals as a reference. Each rat was tested only once to minimize the damage to brain tissue. Rats were anesthetized with a combination of ketamine and xylazine and placed in a stereotaxic apparatus. An incision was made at the midline of the head from slightly posterior to the eyes toward the base of the skull to sufficiently expose the cranium for identification of the landmarks, bregma and lambda*.* The “flat skull” position was achieved by adjusting the incisor piece until bregma and lambda were of similar height (± 0.2 mm), after which bregma was used to establish the stereotaxic position of the red nucleus. A small hole in the cranium was made with a dental drill, and the dura mater was carefully removed. A guide cannula was place 2.0 mm above the left red nucleus, using the following coordinates: 5.8 mm posterior to bregma, 0.7 mm lateral, 7.2 mm below the cortical surface [21]. A stainless steel wire was used to plug the guide cannula that was secured with dental cement and skull screws.

After at least a 24-h recovery time, an injection cannula was insert into the red nucleus through the guide cannula. The location of injection cannula probes was confirmed histologically by an examination of brain slice sections. Fifteen min after the administration of 25 mg kg^−1^ DDD-028 or vehicle, rats received a single microinjection of DTG (Sigma-Aldrich) 5 nmol/0.5 µl and were photographed every 5 min for 30 min. The volume of 0.5 µl was injected over a 1-min period.

### Paw Pressure Test

The nociceptive threshold in the rat was determined with an analgesimeter (Ugo Basile, Varese, Italy) according to the method described by Leighton et al. [22]. Briefly, a constantly increasing pressure was applied to a small area of the dorsal surface of the hind paw using a blunt conical mechanical probe. Mechanical pressure was increased until vocalization or a withdrawal reflex occurred while rats were lightly restrained. Vocalization or withdrawal reflex thresholds were expressed in grams. These limits assured a more precise determination of mechanical withdrawal threshold in experiments aimed to determine the effect of treatments. An arbitrary cutoff value of 100 g was adopted.

### von Frey Test

The animals were placed in 20 × 20 cm Plexiglas boxes equipped with a metallic mesh floor, 20 cm above the bench. A habituation of 15 min was allowed before the test. An electronic Von Frey hair unit (Ugo Basile, Varese, Italy) was used: the withdrawal threshold was evaluated by applying force ranging from 0 to 50 g with an accuracy of 0.2 g. Punctuate stimulus was delivered to the mid-plantar area of each anterior paw from below the mesh floor through a plastic tip, and the withdrawal threshold was automatically displayed on the screen. Paw sensitivity threshold was defined as the minimum pressure required to elicit a robust and immediate withdrawal reflex of the paw. Voluntary movements associated with locomotion were not taken as a withdrawal response. Stimuli were applied on each anterior paw with an interval of 5 s. The measure was repeated 5 times, and the final value was obtained by averaging the 5 measures [23].

### Cold Plate Test

Thermal allodynia was assessed using the cold plate test. With minimal animal-handler interaction, rats were taken from home-cages, and placed onto the surface of the cold-plate (Ugo Basile, Varese, Italy) maintained at a constant temperature of 4 ± 1 °C. Ambulation was restricted by a cylindrical Plexiglas chamber (diameter 10 cm, height 15 cm), with open top. A timer controlled by foot pedal began timing response latency from the moment the mouse was placed onto the cold plate. Pain-related behavior (licking of the hind paw) was determined by recording the time (seconds) of the first sign of licking of the hind paw. The cutoff time of the latency of paw lifting or licking was set at 30 s [24, 25].

### Open Field Test

Rats were placed into the center of the arena, brightly lit (1000 lx). The total distance travelled, the mobility time, the time spent by animals in the center, and in the periphery of the arena within 10 min of observation were recorded [26].

### Tissue Collection

On day 18 (i.e., the end of the experiments on the assessment a protective effect of DDD-028), animals were sacrificed by decapitation. L4-L5 dorsal root ganglia (DRG), sciatic nerve, lumbar spinal cord, and brain were collected, frozen using liquid nitrogen or fixed by immersion in 4% neutral buffered formalin.

### Immunohistochemistry of Brain and Spinal Cord

Formalin fixed cryostat Sects. (10 μm for brain and 5 μm for spinal cord) were incubated for 1 h in blocking solution (Bio-Optica; Italy) at room temperature, and thereafter, sections were incubated for 24 h at 4 °C in PBST containing primary antisera and 5% normal donkey serum. The primary antibody was directed against Iba1 (rabbit antiserum, 1:500; Wako Chemicals, USA [27]) for microglial staining and against glial fibrillary acidic protein (GFAP; rabbit antiserum, 1:500; Dako, USA [28]) for astrocyte staining. After rinsing in PBST, sections were incubated in donkey anti-rabbit IgG secondary antibody labelled with Alexa Fluor 488 or 568 (1:1000, Invitrogen, USA) at room temperature for 1 h.

For all immunohistochemical studies, negative control sections (no exposure to the primary antisera) were processed concurrently with the other sections.

A single optical density value for the dorsal horns in each rat was obtained by averaging the two sides, and this value was compared to the homologous average values from the vehicle-treated animals.

### Quantitative Analyses of Iba1 and GFAP Immunohistochemistry

Images were acquired using a motorized Leica DM6000B microscope equipped with a DFC350FX camera. Morphological examination of microglia and astrocytes was assessed by inspection of at least three fields (× 40 0.75NA objective) in the dorsal horn of the spinal cord and brain areas per section. The full specimen thicknesses were acquired as z-stack series, deconvolved using Huygens Professional software (SVI, The Netherlands) and displayed using ImageJ software.

Quantitative analysis of GFAP- and Iba1-positive cells was performed by collecting at least three independent fields through a × 20 0.5NA objective. GFAP-positive cells were counted using the “cell counter” plugin of ImageJ, whereas Iba1-positive cells were quantified by means of the automatic thresholding and segmentation features of ImageJ. Quantification of GFAP signal in immunostained sections was also performed using FIJI software by automatic thresholding images with the aid of the “Moments” algorithm, which delivered the most consistent pattern recognition across all acquired images.

### Carbonylated Protein Evaluation

Sciatic nerve and DRGs protein extracts were quantified by bicinchoninic acid. Five micrograms of each sample were denatured by 6% SDS and derivatized by 15-min incubation with 2, 4 dinitrophenyl hydrazine (DNPH; Sigma-Aldrich, Italy) at room temperature. Samples were separated on a 4–12% sodium dodecyl sulfate (SDS)-polyacrylamide gel by electrophoresis and transferred onto nitrocellulose membranes (Biorad, Italy). Membranes were blocked with 1% bovine serum albumin (BSA) in phosphate-buffered saline (PBS) containing 1% Tween 20 (PBST) and then probed overnight with primary antibody specific versus DNPH (Sigma-Aldrich, Italy) 1:5000 in PBST/1% BSA. After washing with PBST, the membranes were incubated for 1 h in PBST containing the appropriate horseradish peroxidase-conjugated secondary antibody (1:5000; Cell Signalling, USA) and again washed. ECL (Pierce, USA) was used to visualize the peroxidase-coated bands. Densitometric analysis was performed using the “Image J” analysis software, and the density of all bands displayed in the lane is reported as a mean. Ponceau-stained membranes were used as loading control [34].

### Catalase Activity

Enzymatic activity in both DRGs and sciatic nerve was measured in PBS using the homogenated tissues: the suspension was sonicated in ice using three 10 s bursts at high intensity with a 10-s cooling period between each burst and then centrifuged (13.000 × *g* for 15 min at 4 °C). Catalase activity was measured in the supernatant by Amplex Red Catalase Assay Kit (Invitrogen, Monza, Italy) following the manufacturer’s instructions. Protein concentration was quantified by bicinchoninic acid assay (Sigma-Aldrich, Milan, Italy). Catalase activity for each sample was normalized to protein concentration. Control conditions in the absence of treatment were set as 100% [29].

### Statistical Analysis

The results were expressed as mean ± SEM, and data were analyzed using the “Origin 9.1” software. Statistical analysis was performed using one-way ANOVA followed by post hoc Bonferroni’s significant difference procedure. *P* values of less than 0.05, 0.01, or 0.001 were considered significant. All data were collected by an observer who was blinded to the treatments.

### Results

**Fig. 2:**
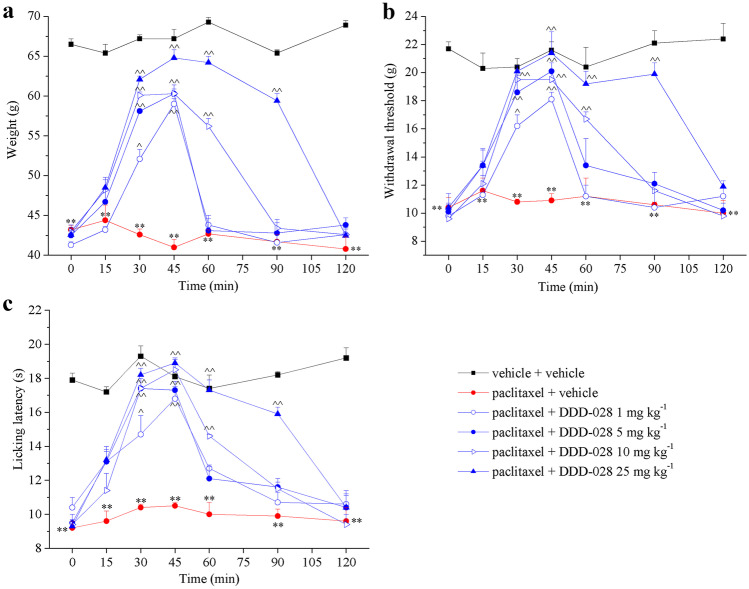
Effect of single DDD-028 administrations on pain behavior induced by paclitaxel. Sensitivity to a noxious mechanical stimulus as measured by the paw pressure test (**a**). Pain threshold to a non-noxious mechanical stimulus as measured by the von Frey test (**b**). Pain threshold to a non-noxious thermal stimulus as measured by the cold plate test (**c**). Paclitaxel (2.0 mg kg^−1^, i.p.) was administered on four days (1, 3, 5, and 8). Starting from day 10, DDD-028 was acutely per os administered (1–25 mg kg^−1^) and measurements assessed before treatment and 15, 30, 45, 60, 90, and 120 min after injection. Results were expressed as mean ± SEM of 8 rats analyzed in 2 different experimental sets. **P < 0.01 vs vehicle + vehicle; ^^*P* < 0.01 vs paclitaxel + vehicle

Acute analgesic effects (mechanical hyperalgesia, thermal and mechanical allodynia) of DDD-028 are shown in Fig. 2a–c. For these studies, DDD-028 was acutely per os administered when neuropathy was well established (day 10), 48 h after the last chemotherapeutic drug injection. On day 10, paclitaxel-treated rats showed a significant reduction of the weight tolerated on posterior paws with respect to the control animals (43.2 ± 0.5 g vs 66.5 ± 0.7 g, respectively) (Paw pressure test; Fig. 2a). Increasing doses of DDD-028 (1–25 mg kg^−1^) reduced mechanical hypersensitivity in a dose-dependent manner starting 30 min after treatment. The highest dose of DDD-028 (25 mg kg^−1^) completely abrogated paclitaxel-induced mechanical hyperalgesia with maximum analgesic effect occurring at 30 min. The effect persisted for at least 90 min and then vanished 120 min after treatment. Strong, albeit slightly reduced, analgesic effect was also observed at the doses of 10, 5, and 1 mg kg^−1^, but the duration of the analgesic effect was reduced from about 30–90 min at 25 mg kg^−1^ to about 30–60 min at 1 mg kg^−1^ (Fig. 2a). In the same way, acute administration of DDD-028 counteracted paclitaxel-induced mechanical allodynia in a dose-dependent manner in the von Frey test (Fig. 2b). The highest dose again showed a complete reversal of paclitaxel-induced neuropathy with a long-lasting effect starting from 30 up to 90 min after treatment. All the lower doses of DDD-028 displayed a shorter anti-hyperalgesic efficacy (Fig. 2b). Finally, DDD-028 also counteracted paclitaxel-induced thermal allodynia in a dose-dependent manner in the Cold Plate test (Fig. 2c). As shown in Fig. 2c, paclitaxel alone significantly enhanced the sensitivity to cold after 10 days of treatment. Thermal allodynia was fully alleviated by DDD-028 (25 mg kg^−1^) administration. The result obtained with the higher dose was more effective and long-lasting with respect to the lower doses that were capable anyway to reach the statistical significance peaking 45 min after administration (Fig. 2c).

**Fig. 3:**
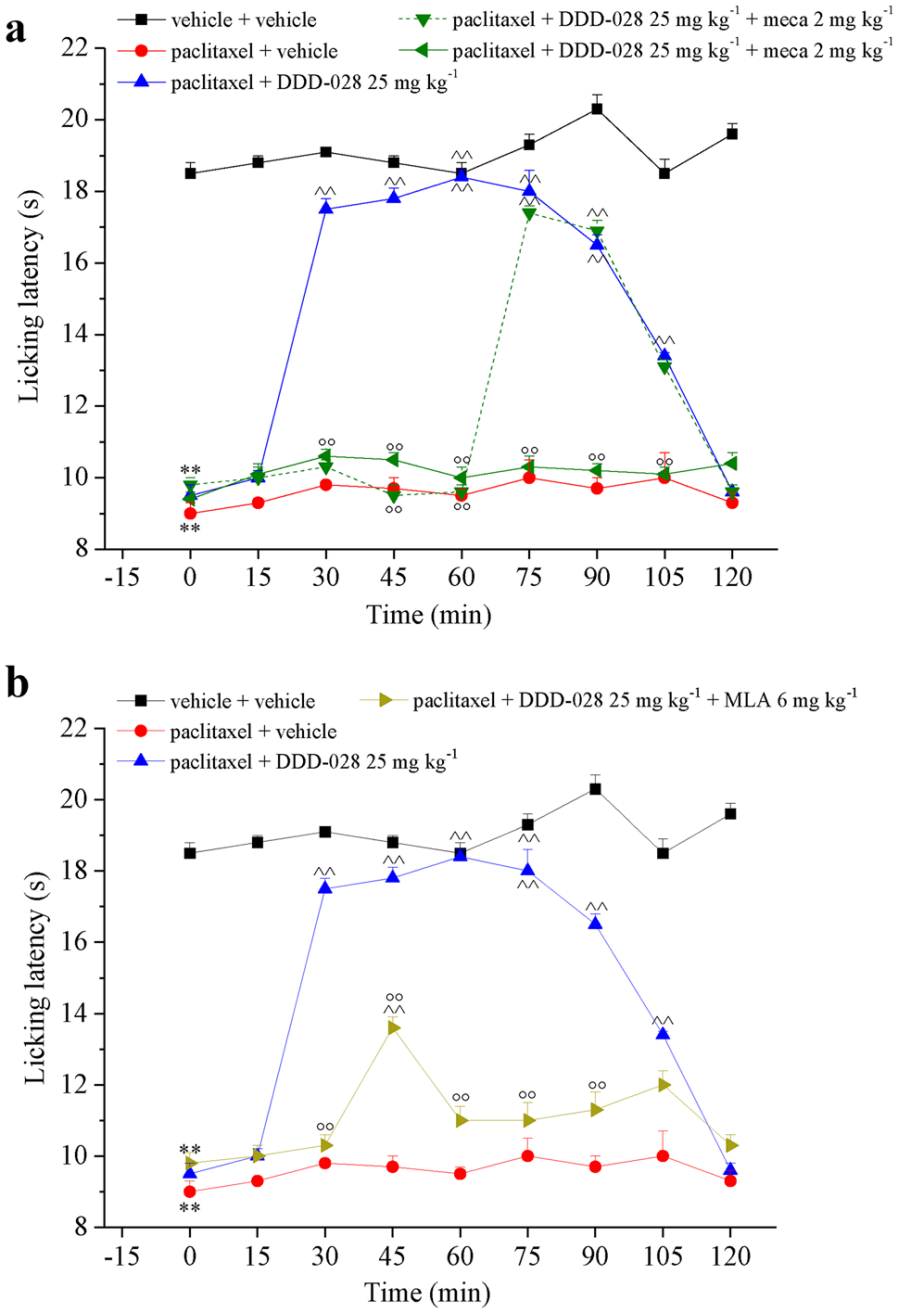
Study of DDD-028 pharmacodynamic profile. The nAChRs (**a**) and α7 nAChR (**b**) involvement in DDD-028 effects. Pain was induced by repeated treatment with paclitaxel. The hypersensitivity to a cold stimulus was measured by the Cold plate test. DDD-028 was administered per os at 25 mg/kg. The nAChR antagonist MECA (2 mg kg^−1^) was administered intraperitoneally 15 min before DDD-028 administration. In a separate experiment, mecamylamine was administered for a second time 45 min after DDD-028. The α7 nAChR antagonist MLA (6 mg kg^−1^) was administered intraperitoneally 15 min before DDD-028 injection. Results were expressed as mean ± SEM of 8 rats analyzed in 2 different experimental sets. **P < 0.01 vs vehicle + vehicle; ^^*P* < 0.01 vs paclitaxel + vehicle; °°*P* < 0.01 vs paclitaxel + DDD-028

To study the pharmacodynamics of DDD-028, three possible targets were hypothesized based on previous evidence obtained by binding studies [5]: particularly the nicotinic receptor (nAChR) of the cholinergic system, the voltage-gated potassium channel subtype Kv7, and sigma 1 (σ_1_) and 2 (σ_2_) receptors. In the cold plate test, as shown in Fig. 3a, DDD-028 (25 mg kg^−1^, per os) increased the licking latency of paclitaxel-treated animals starting 15 min after administration and lasting up to 105 min. Pre-treatment of the animals with the nAChR antagonist MECA (2 mg kg^−1^, i.p.) 15 min before DDD-028 administration completely abolished the pain-relieving effect of the compound up to 60 min. However, when MECA was administered the second time at 45 min after DDD-028 treatment, the effect of DDD-028 was entirely blocked for all the times observed.

The anti-hyperalgesic effect induced by DDD-028 was also substantially reduced by the pre-treatment with the α7 nAchR antagonist MLA (6 mg kg^−1^, i.p.) (Fig. 3b), which suggests that this receptor subtype is very likely involved in the mechanism of action of DDD-028.

The relevance of the Kv7 channels in the DDD-028 mechanism was studied by using the selective Kv7 antagonist XE991 (1 mg/kg, i.p.) (Supplementary Fig. S1a). XE991 administered 15 min before DDD-028 was not able to alter the efficacy of DDD-028 over the time of observation (Supplementary Fig. S1a).

Finally, based on previous studies, that σ_1_ receptor is involved in the pain pathway, DDD-028 was subjected to the modulation of the σ receptor-mediated neck dystonia [20]. Accordingly, the σ agonist DTG was injected in the red nucleus. DTG induced postural changes characterized by a marked deviation in the head angle (neck dystonia), peaking at 25–30 min after microinjection (Supplementary Fig. S1b). DDD-028 (25 mg kg^−1^) was administered per os 15 min before DTG infusion without preventing DTG-induced neck dystonia (Supplementary Fig. S1b).

**Fig. 4:**
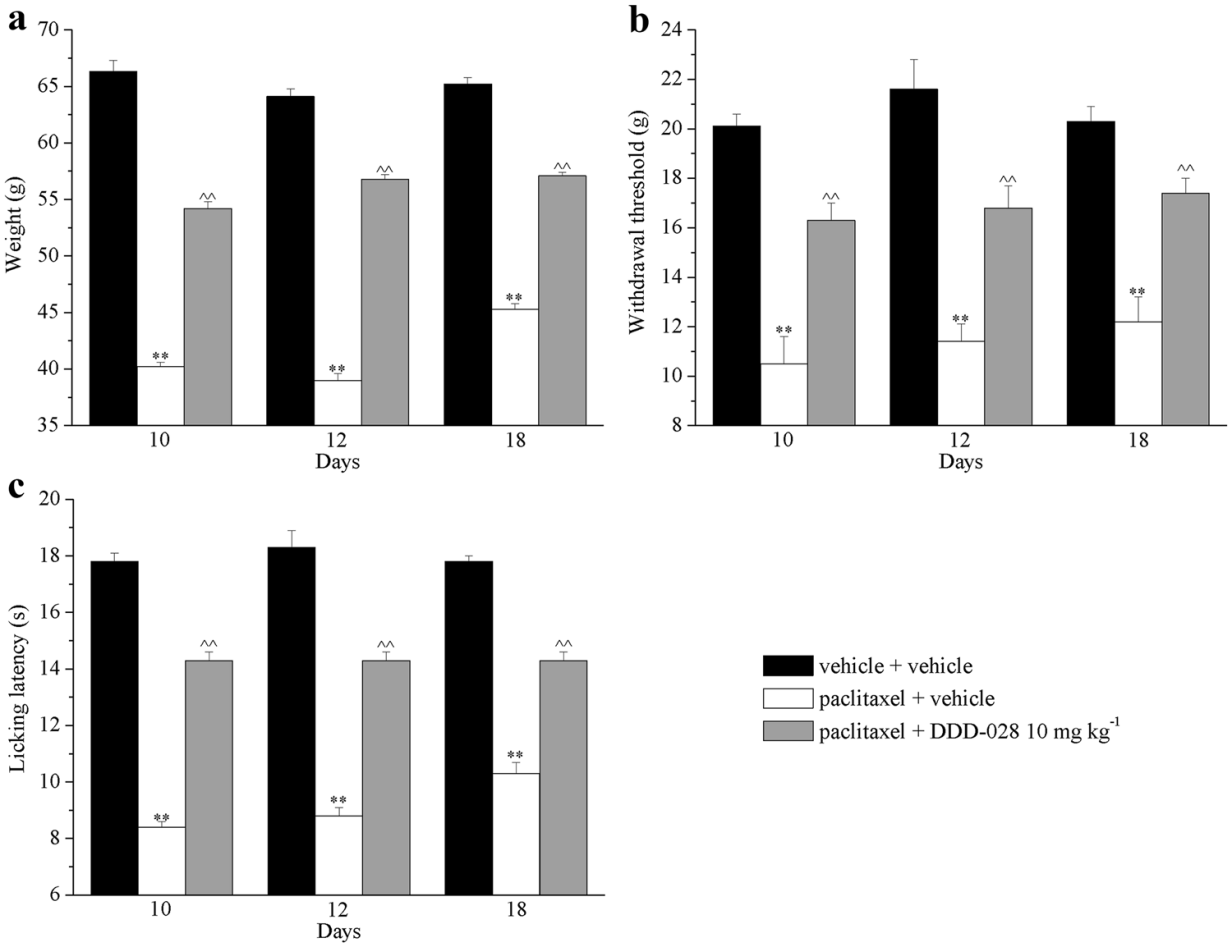
Effects of repeated administration of DDD-028 on pain behavior induced by paclitaxel. Sensitivity to a noxious mechanical stimulus as measured by the paw pressure test (**a**). Pain threshold to a non-noxious mechanical stimulus as measured by the von Frey test (**b**). Pain threshold to a non-noxious thermal stimulus as measured by the cold plate test. Behavioral tests were performed on days 10, 12, and 18 after the beginning of paclitaxel and DDD-028 administrations, 24 h after the last treatment. Paclitaxel (2.0 mg kg^−1^, i.p.) was administered on four days (1, 3, 5, and 8) while DDD-028 (10 mg kg^−1^, p.o.) was daily administered, starting from day 1 of paclitaxel injection. Results were expressed as mean ± SEM of 8 rats analyzed in 2 different experimental sets. **P < 0.01 vs vehicle + vehicle; ^^*P* < 0.01 vs paclitaxel + vehicle

Thereafter, to assess the protective profile of DDD-028, the compound was subjected to repeated administrations over an 18-day period. Paclitaxel-treated animals were administered daily with DDD-028 (10 mg kg^−1^, p.o.) starting from the same day of paclitaxel injection. The response to mechanical noxious stimulus was measure on days 10, 12, and 18, 24 h after the last treatment. As shown in Fig. 4a, DDD-028 significantly increased the pain threshold of paclitaxel-injected rats at all-time points considered without development of tolerance to the anti-hypersensitivity effect (Fig. 4a). Repeated administration of DDD-028 induced similar results in reducing paclitaxel-induced mechanical and thermal allodynia as shown by Fig. 4b, c. In both measurements, DDD-028 increased the withdrawal latency and the licking latency of the animals at all-time points as evidenced by the Von Frey and the cold plate tests, respectively. The efficacy of DDD-028 was not different among 30 min and 24 h after treatment suggesting a stable improvement of the pain threshold (Supplementary Fig. S2). Moreover, non-reflexive measures, as evaluation of spontaneous pain, were also performed by the Open field test. On day 18, the total distance travelled, the time spent in the center and in the periphery of the arena and the mobility time of each animal was recorded. No statistically significant differences between groups were found analyzing these parameters (Supplementary Table S1).

**Fig. 5:**
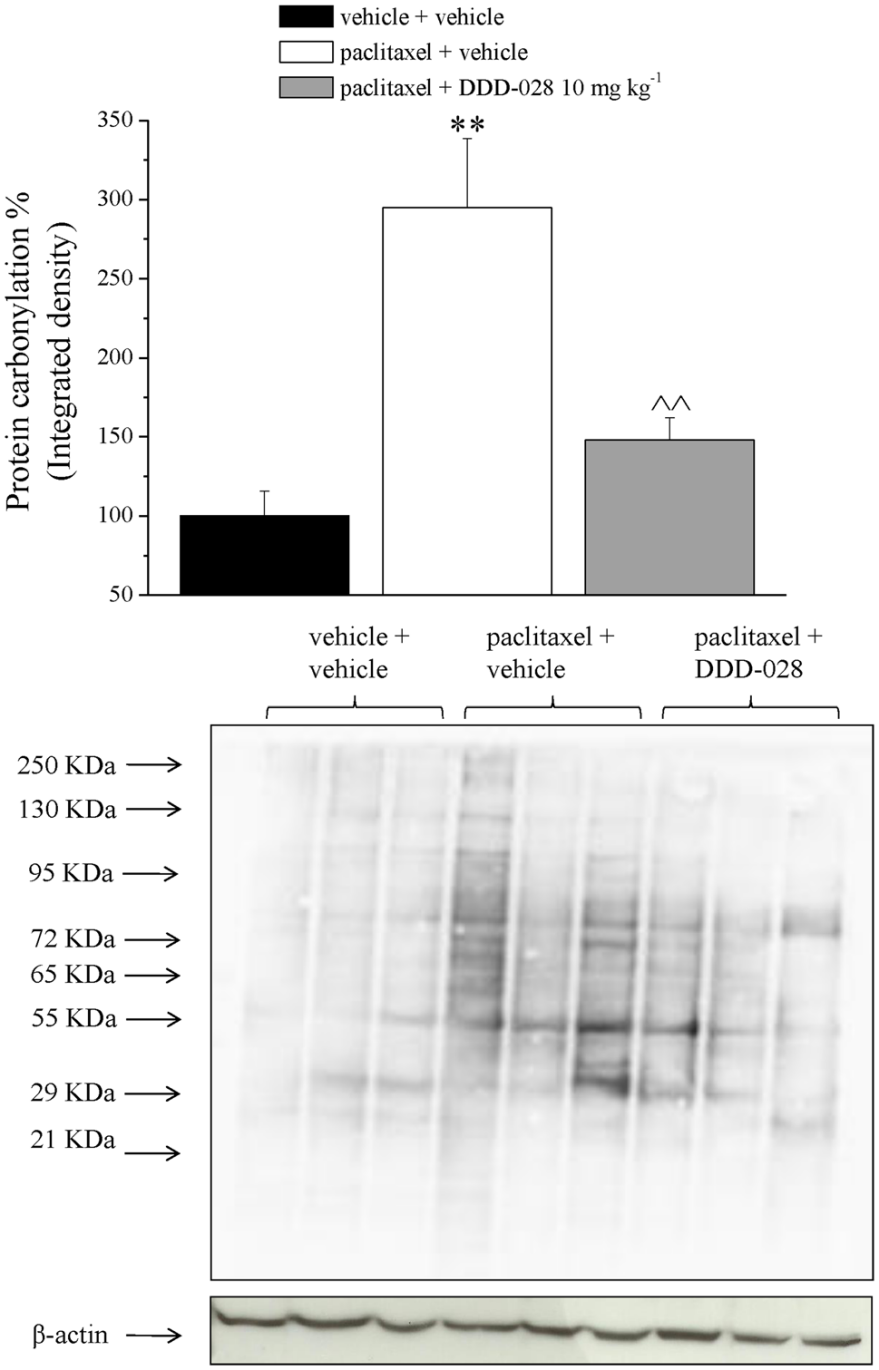
Carbonylated protein. Dorsal root ganglia. Densitometric analysis, data were normalized on the expression of beta-actin as housekeeping and expressed as mean ± SEM of 6 samples from 6 different animals analyzed twice. Representative western blot was also showed (3 samples of each treatment are shown). **P < 0.01 vs vehicle + vehicle; ^^*P* < 0.01 vs paclitaxel + vehicle

To evaluate the capability of DDD-028 to intervene against direct damages and against the maladaptive plasticity of the nervous system induced by paclitaxel, nervous tissues (brain, spinal cord, DRGs, and sciatic nerve) were collected and analyzed at the end of the repeated treatment with the compound (day 18). Oxidative stress, a typical signature of chemotherapy-induced neurotoxicity [30, 31], was measured in the peripheral nervous system. As shown in Fig. 5, paclitaxel induced an oxidative damage of DRG as indicated by three-fold increase in carbonylation of proteins. Treatment with DDD-028 resulted in a significant prevention of the damage as evidenced by protein carbonylation values similar to the control group (Fig. 5, densitometric analysis and representative plot). The sciatic nerve was not affected by this kind of oxidative damage suggesting a stronger toxicity on DRG (Supplementary Fig. S3, densitometric analysis and representative plot).

**Fig. 6:**
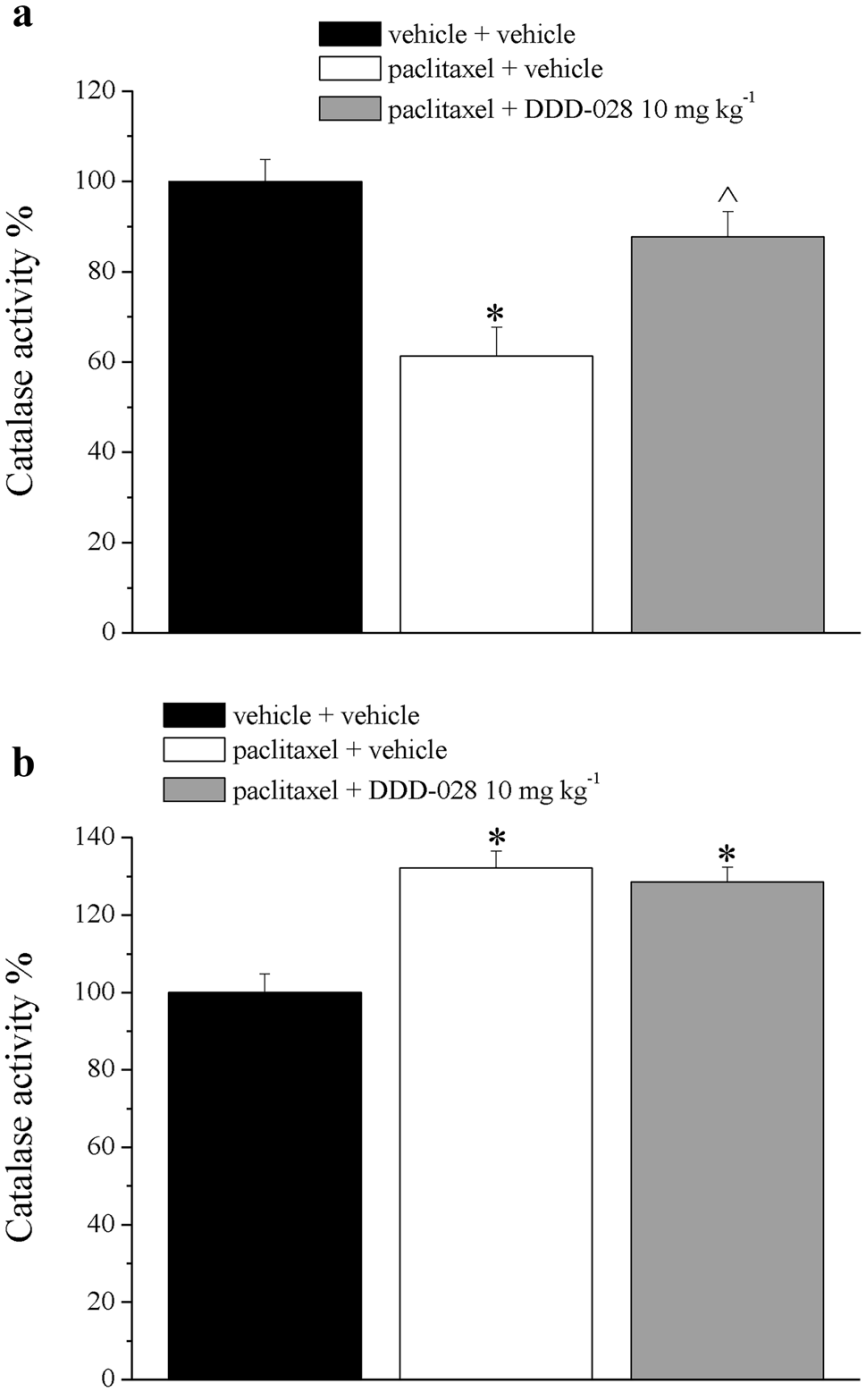
Catalase activity. Dorsal root ganglia (**a**) and sciatic nerve (**b**) were analyzed. Enzymatic activity was expressed as percentage of control (vehicle + vehicle was considered as 100%). Data were expressed as mean ± SEM of 6 samples from 6 different animals analyzed in triplicate. *P < 0.05 vs vehicle + vehicle; ^*P* < 0.05 vs paclitaxel + vehicle

Catalase activity, another marker of the state of functionality of peroxisome, is shown in Fig. 6a; paclitaxel-treated animals showed a 40% decrease of catalase activity at the DRG, suggesting an impairment of the organelle. Treatment with DDD-028 significantly rescued the enzymatic activity. On the other hand, the sciatic nerve, which is damaged to a lesser extent than DRG induced by paclitaxel as indicated in Fig. 5b, showed an increase of catalase activity that can be explained as a detoxifying effort of cells of the sciatic nerve to counteract paclitaxel toxicity. DDD-028 did not interfere in this survival mechanism (Fig. 6b).

**Fig. 7:**
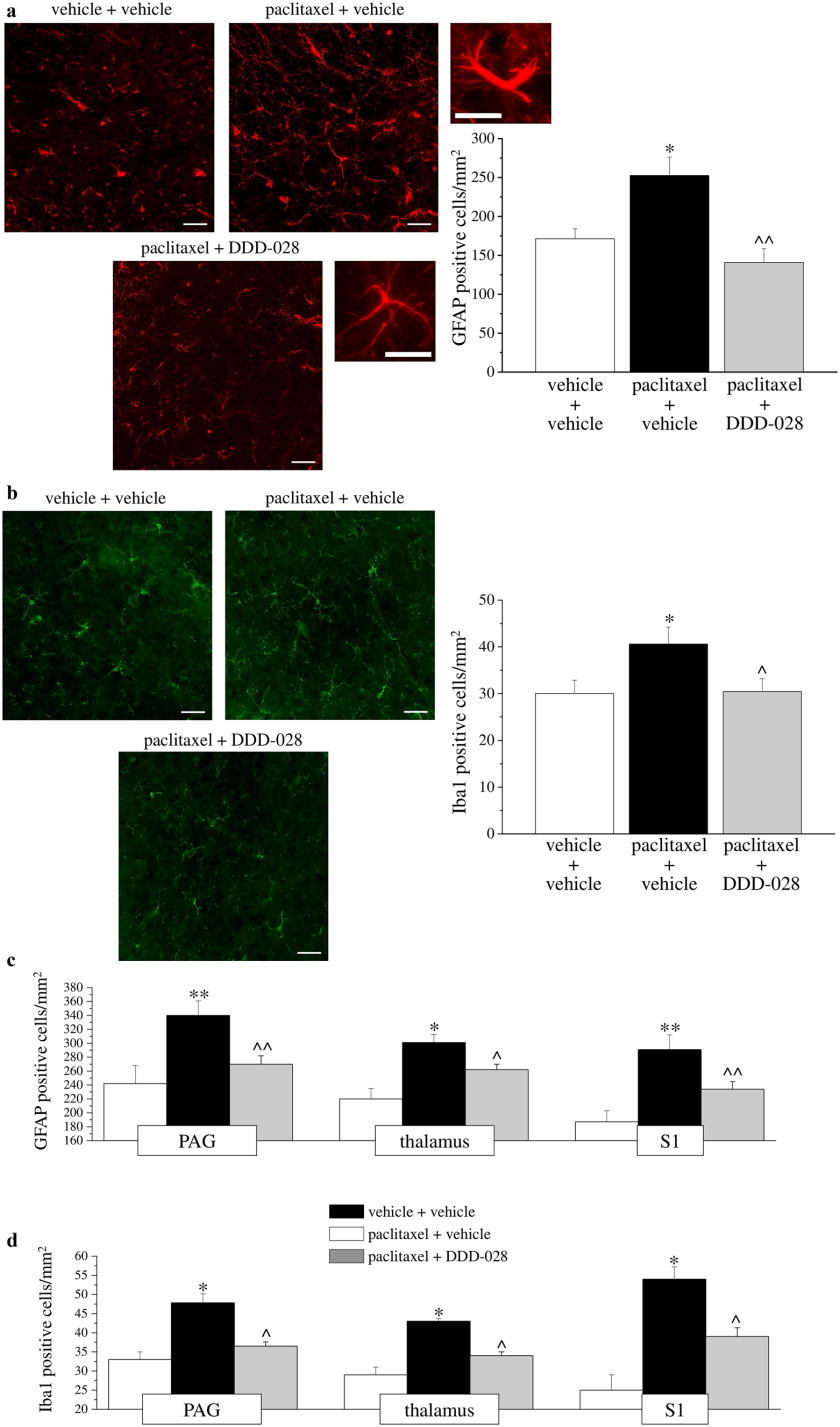
Glial cells analysis. Spinal cord. Astrocytes (**a**) were studied by immunohistochemistry performed with a GFAP antibody. Representative image of the dorsal horn (lumbar level) at × 20 magnification; × 40 images were shown to highlight morphological alterations. Quantitative analysis was reported as number of GFAP-positive cells, and data were expressed as mean ± SEM of 3 different fields of 3 specimens for each of 6 samples from 6 different animals. *P < 0.05 vs vehicle + vehicle; ^*P* < 0.05 and ^^*P* < 0.01 vs paclitaxel + vehicle. Microglia (**b**) was studied by immunohistochemistry performed with a Iba1 antibody. Representative image of the dorsal horn (lumbar level) at × 20 magnification. Quantitative analysis was reported as number of Iba1-positive cells, and data were expressed as mean ± SEM of 3 different fields of 3 specimens for each of 6 samples from 6 different animals. *P < 0.05 vs vehicle + vehicle; ^*P* < 0.05 vs paclitaxel + vehicle. Brain. Astrocytes (**c**) and microglia (**d**) were studied by immunohistochemistry performed with GFAP and Iba1 antibodies, respectively. Analysis was performed on periaqueductal grey (PAG), thalamus, and somatosensory area 1 (S1). Quantitative analysis was reported as number of GFAP- and Iba1-positive cells, and data were expressed as mean ± SEM of 3 different fields of 3 specimens for each of 6 samples from 6 different animals. *P < 0.05 vs vehicle + vehicle; ^*P* < 0.05 vs paclitaxel + vehicle

To determine whether neurochemical reorganization in the spinal cord occurs following DDD-028 repeated treatment, we examined the lumbar spinal cord sections by immunohistochemistry using antibodies against GFAP and Iba1 to label astrocytes and microglia, respectively, which are non-neuronal cells strongly involved in chemotherapy-induced neuropathic pain [32, 33]. Astrocyte activation was measured as an increase in the number of GFAP-expressing cells in the dorsal horn of the spinal cord of treated rats. GFAP-positive cell number in superficial laminae of paclitaxel-treated rats was significantly greater than the vehicle-treated cell number at day 18 (Fig. 7a). Moreover, spinal astrocytes presented altered morphology showing hypertrophy of the cell *body* and processes (Fig. 7a). Animals treated with paclitaxel + DDD-028 showed a lower number of astrocytes characterized by a reactive phenotype. Cell density increase was also significantly prevented (Fig. 7a).

Microglia activation was measured by the quantification of Iba1-positive cells in the spinal cord of treated rats. On day 18, paclitaxel treatment produced increased density of Iba1-positive cells in the dorsal horns of the lumbar spinal cord (Fig. 7b). However, no hypertrophy of this type of glia cells was observed, and microglia possessed a highly ramified morphology similar to microglia in saline rats. On the other hand, day 18 can be considered a late phase for microglia activation, that is, according to the literature, strongly involved in the first days of treatment. Animals treated with paclitaxel + DDD-028 showed a significant prevention of microglia activation (Fig. 7b).

To probe the effect of DDD-028 in the brain regions involved in pain sensation, a topographic analysis of microglia and astrocyte cells in three areas, the periacqueductal gray (PAG; involved in endogenous pain modulatory system), the thalamus and the somatosensory cortex (S1) were examined. As shown in Fig. 7c, d, paclitaxel induced a significant numerical increase of both glial cell populations in brain areas, but a higher astrocyte activation was observed. DDD-028 reduced both microglia and astrocyte cell number increase (numerical activation) in PAG, thalamus and S1 (Fig. 7c, d). The morphological analysis of Iba1 and GFAP immunopositive brain cells did not reveal any sign of reactivity (usually the activated microglia cells are characterized by a ramified aspect with a cell body that was always the most intensely labeled and with slender and radially projecting processes; activated astrocytes showed multiple branched processes extending in different directions from an elongated cell body).

## Discussion

The results of this study highlighted that DDD-028, a pentacyclic pyridoindole heterocycle, was able to exert anti-neuropathic and protective effects in the paclitaxel-induced neuropathic pain animal paradigm. Pain relieving efficacy was mediated by the α7 nAChR subtype.

In the last decade, early detection of tumor with concomitant success of anticancer therapies has led to an increase in cancer survival rate. Unfortunately, cancer chemotherapy is also beset with iatrogenic adverse effects, and neuropathies [34] are unavoidable toxicity of chemotherapy treatments that are endured by patients in exchange for a life extension offered by these drugs. Moreover, no preventive or therapeutic options are currently available for the management of neuropathic pain [35], and new disease-modifying approaches to treat neuropathies remains a critical unmet need.

DDD-028 emerged from binding and functional studies as promising candidate. The Lipinski-Veber rules for drug-like properties [11], and the central nervous system multiparameter optimization (CNS MPO) [10] value of 4.5 places it in the high desirability range for drugs that are purported to target the CNS. In vivo experiments, DDD-028 displayed potent acute anti-hyperalgesic activity in both of the widely used rodent models of neuropathic pain such as chronic constriction injury (CCI) and spinal nerve ligation (SNL) at oral doses between 1 and 5 mg kg^−1^ [6]. Moreover, its efficacy was also confirmed in an inflammatory pain model such as that induced by the intra-articular injection of the complete Freund’s adjuvant (CFA) [6]. Preliminary pharmacokinetic study in rats indicated that DDD-028 elicited pain relief at low peak plasma concentration (C_max_, 1.91 ng/mL), which suggests a wide safety window (c.a. ~ 500 based on hERG binding) for the expected dose regimen. In our work, we explored the pain relieving properties of the molecule in neuropathic pain condition induced by the antitumor drug paclitaxel. DDD-028 was able to recover, dose dependently, paclitaxel-induced hypersensitivity as well as to prevent the development of neuropathic symptoms when was co-administered with the chemotherapeutic drug. Moreover, the sub-chronic administration of DDD-028 did not develop tolerance to the antinociceptive effect exerted in paclitaxel-treated mice, in contrast to other known antinociceptive or analgesic drugs, such as morphine, which induces tolerance after repeated administration both in naïve animals [31, 36] and in mice and rats treated with paclitaxel representing one of the most limiting side effects of opioids [37, 38]. It has been previously reported that DDD-028 dose not bind to any of the opioid, cannabinoid, histamine, or dopamine receptors [5]. As it binds to σ_1-_σ_2_ receptors with moderate affinity, we tested DDD-028 in vivo because of the relevant role of σ antagonists in pain control [39, 40]. As mentioned before, to evaluate the σ-mediated effect, we measured the neck dystonia induced by the selective σ agonist DTG after injection in the red nucleus [20]: DTG efficacy was unmodified by DDD-028. Two other pharmacodynamic mechanisms were also analyzed. Opening of Kv7 may regulate neuron excitability showing efficacy against neuropathic pain [18, 19], but the anti-hypersensitive effect of DDD-028 was not blocked by the Kv7 blocker XE991. Finally, nAChRs, a family of channel receptor strongly implied in pain regulation and candidate to be interesting drug targets [41]. The acute effect of DDD-028 was fully blocked by the both nAChR non-specific antagonist MECA, and by the selective α7 nAChR antagonist MLA. In several models of neuropathic pain (induced by toxic, traumatic, or metabolic events), the α7 subtype stimulation emerged as relevant target for both pain control and neuroprotection [42–45]. In this context, it appears that α7 nAChR may be involved in the mechanism of action of DDD-028, and the identification of precise downstream pathway is under investigation. Regarding neuroprotective properties, it is well established that in PNS, in general, and in DRGs, in particular, are the first targets for the dysregulation of antioxidant enzymes following paclitaxel treatment [46, 47]. Among the multiplicity of the pathophysiologic mechanisms, this oxidative stress plays a crucial role in the generation of paclitaxel-induced neuropathy.

ROS act in normal cellular processes and the concentration of these compounds is controlled by the antioxidant system that involves numerous non-enzymatic molecules and enzymes such as superoxide dismutase (SOD) and catalase. The unbalance of redox mechanisms provokes alterations to proteins, lipids, and DNA as highlighted also in chemotherapy-induced neuropathies [30, 48]. As a consequence, antioxidants were investigated as a possible treatment. TEMPOL (4-hydroxy-2,2,6,6-tetramethyl piperidinoxyl), a superoxide dismutase mimetic, inhibited the development and maintenance of paclitaxel-induced mechanical hypersensitivity [49], phenyl-N-t-butyl nitrone (PBN), a non-specific ROS scavenger, inhibited the development of paclitaxel-induced mechanical hypersensitivity [50] while another SOD mimetic, MnL4, reduced mechanical hyperalgesia and thermal allodynia induced by oxaliplatin administration [51].

DDD-028 was able to prevent the development of hypersensitivity, and to reduce paclitaxel-related damages to PNS and CNS when repeatedly administered with the anticancer drug. DDD-028 demonstrated detoxifying properties as indicated by the enhancing the activity of the peroxisomal enzyme, catalase, and reducing the protein oxidation in DRGs.

The protective properties of DDD-028 are extended also to CNS, where a complex maladaptive response of neuronal and non-neuronal cells orchestrates the chronicization of pain. Glial cells, in particular, have been shown to contribute to the development of chronic pain in various conditions including surgery, inflammation, and nerve injury [33, 52]. Pharmacological treatments including minocycline [53] and fluorocitrate [32, 54] were able to prevent glial activation and reduce neuropathic pain. According to [55] and to [54], we observed an increased number of Iba1- (microglia) and GFAP- (astrocytes) positive cells in the dorsal horn of the lumbar spinal cord after a cumulative dose of 8 mg kg^−1^ paclitaxel indicating an astrocytes activation on day 18 after the beginning of treatment. This increase was also detected in supraspinal pain stations like PAG, thalamus, and somatosensory cortex 1. Microglia and astrocyte activation in all these areas was prevented by DDD-028 repeated treatment. Although it is generally believed that paclitaxel does not penetrate blood–brain barrier [56, 57], low concentrations of paclitaxel can be detected in spinal cord after systemic treatment and this could be the explanation regarding the central effects recorded on astrocytes [58]. Thus, further study is needed to evaluate whether the activation of spinal astrocytes is induced by the direct effect of paclitaxel (probably due to its transport along the centrifugal and centripetal branches of the DRG neuron axons, as suggested by Cavaletti et al. [58]) or through its effects on peripheral targets.

In summary, DDD-028 seems to be a promising candidate for the management of paclitaxel-induced neuropathy. Its anti-hyperalgesic effect is mediated by the nicotinic system, particularly by the α7 nAChR subtype. Further, DDD-028’s profile appears to be a disease-modifying agent that is able to counteract oxidative damages of PNS and to reduce the maladaptive plasticity of spinal and supraspinal glial cells.

## Supplementary Information

Below is the link to the electronic supplementary material.

Supplementary file1 (PDF 2058 KB)

Supplementary file2 (PDF 1281 KB)

Supplementary file3 (PDF 1259 KB)

Supplementary file4 (PDF 2216 KB)

Supplementary file5 (PDF 2248 KB)

Supplementary file6 (PDF 2268 KB)

Supplementary file7 (PDF 2372 KB)

Supplementary file8 (PDF 2283 KB)

Supplementary file9 (PDF 2091 KB)

Supplementary file10 (PDF 2165 KB)

Supplementary file11 (DOCX 358 KB)
