# Supplementary material for: Pain Relieving and Neuroprotective Effects of Non-opioid Compound, DDD-028, in the Rat Model of Paclitaxel-Induced Neuropathy
Source: Neurotherapeutics. 2021 Jul 26;18(3):2008–20. doi: 10.1007/s13311-021-01069-8 (PMC8608957; doi:10.1007/s13311-021-01069-8)
Supplement: Supplementary file 11 — Supplementary file11 (DOCX 358 KB) [file 13311_2021_1069_MOESM11_ESM.docx]

**Supplementary materials for**

**Pain relieving and neuroprotective effects of non-opioid compound, DDD-028, in the rat model of paclitaxel-induced neuropathy**


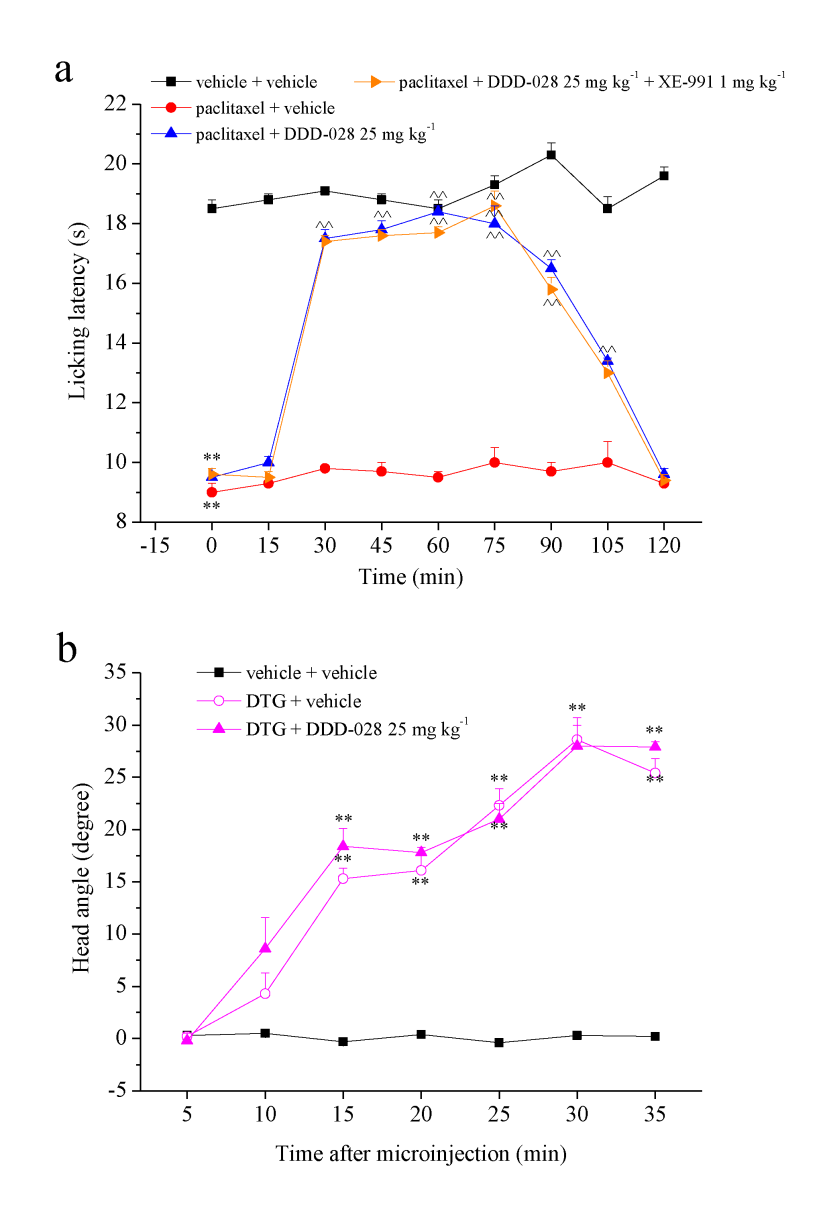


**Supplementary Fig. 1**. Study of the Kv7 potassium channels involvement in DDD-028 effects (b). Pain was induced by repeated treatment with paclitaxel. The hypersensitivity to a cold stimulus was measured by the Cold plate test. DDD-028 was administered *per os* at 25 mg kg^-1^. The Kv7 antagonist XE991 (1 mg kg^-1^) was administered intraperitoneally 15 min before DDD-028 administration. Results were expressed as mean ± S.E.M. of 8 rats analyzed in 2 different experimental sets. **P<0.01 *vs* vehicle + vehicle; ^^P<0.01 *vs* paclitaxel + vehicle.

Study of the σ receptors involvement in DDD-028 effects (c). Neck dystonia was induced by the microinjection of the σ receptors agonist DTG (0.5 nmol/1 μl) in the red nucleus. DDD-028 (25 mg kg^-1^) was administered per os 15 min before DTG infusion. The head angle deviation was measured over time. Results were expressed as mean ± S.E.M. of 8 rats analyzed in 2 different experimental sets. **P<0.01 *vs* vehicle + vehicle.

**Supplementary Fig. S2.** Effects of repeated administration of DDD-028 on pain behavior induced by paclitaxel. Sensitivity to a noxious mechanical stimulus as measured by the Paw Pressure test (a). Pain threshold to a non-noxious mechanical stimulus as measured by the Von Frey test (b). Pain threshold to a non-noxious thermal stimulus as measured by the Cold Plate test. Behavioural tests were performed on days 10, 12 and 18 after the beginning of paclitaxel and DDD-028 administrations, 30 min after the daily treatment. Paclitaxel (2.0 mg kg^-1^, i.p.) was administered on four days (1, 3, 5 and 8) while DDD-028 (10 mg kg^-1^, p.o.) was daily administered, starting from day 1 of paclitaxel injection. Control animals were treated with vehicles. Results were expressed as mean ± S.E.M. of 8 rats analyzed in 2 different experimental sets. **P<0.01 *vs* vehicle + vehicle; ^^P<0.01 *vs* paclitaxel + vehicle


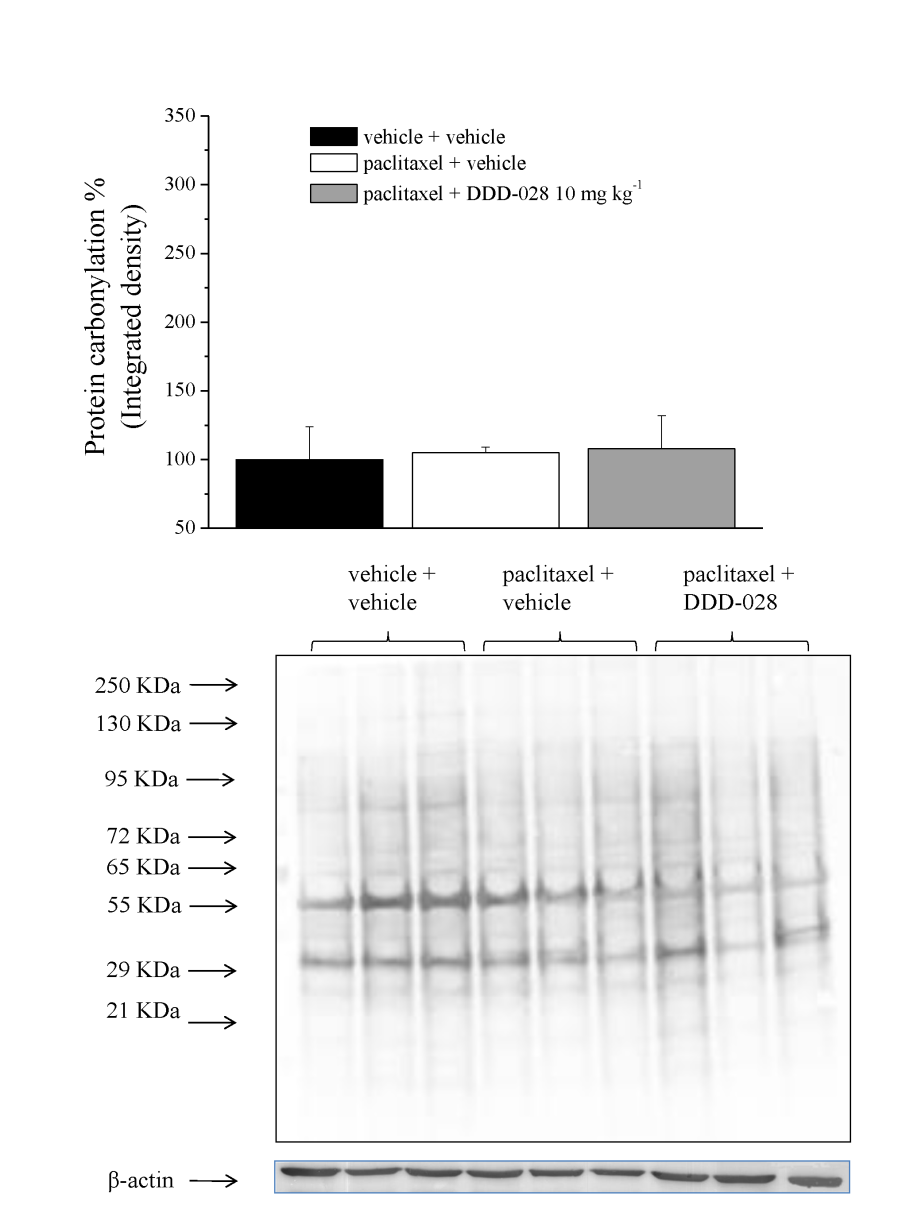


**Supplementary Fig. 3.** Carbonylated protein. Sciatic nerve. Densitometric analysis, data were normalized on the expression of beta-actin as housekeeping and expressed as mean ± S.E.M. of 6 samples from 6 different animals analyzed twice. Representative western blot was also showed (3 samples of each treatment are shown). **P<0.01 *vs* vehicle + vehicle; ^^P<0.01 *vs* paclitaxel + vehicle.

|  | Day 18 | | | |
| --- | --- | --- | --- | --- |
| Treatment | Distance travelled (m) | Mobility time  (s) | Time spent in the center (s) | Time spent in the periphery (s) |
| vehicle + vehicle | 38.9 ± 6.8 | 341.5 ± 32.4 | 9.1 ± 2.4 | 581.2 ± 5.5 |
| paclitaxel + vehicle | 41.4 ± 5.0 | 389.4 ± 22.8 | 9.1 ± 1.9 | 567.1 ± 6.8 |
| paclitaxel + DDD-028 | 33.6 ± 4.5 | 345.4 ± 24.4 | 15.4 ± 4.1 | 581.6 ± 4.2 |

**Supplementary Table S1: Open field test**

Open field test was assessed as a non-reflexive measure for the evaluation of the effect of DDD-028 on paclitaxel-induced neurotoxicity. Paclitaxel (2.0 mg kg^-1^) was intraperitoneally injected on four alternate days (1, 3, 5 and 8), DDD-028 (10 mg kg^-1^) was daily orally administered starting from the first day of paclitaxel up to the end of the experiment (day 18). The open field was performed on day 18, 24 h after the last administration of DDD-028 evaluating the total distance travelled (m), the mobility time (s), the time spent in the center of the arena (s) and the time spent in the periphery of the arena (s) during 10 min of observation. Control animals were treated with vehicles.
